# 3-Phenyl-1,5-di-2-thienylpentane-1,5-dione

**DOI:** 10.1107/S1600536808009884

**Published:** 2008-04-16

**Authors:** Tuan-Jie Meng, Xian-Qiang Huang, Qing-Peng He, Jian-Yong Wang

**Affiliations:** a Department of Chemistry, Shangqiu Normal University, Shangqiu Henan Province 476000, People’s Republic of China; b Department of Chemistry, Liaocheng University, Liaocheng 252059, People’s Republic of China

## Abstract

The asymmetric unit of the title compound, C_19_H_16_O_2_S_2_, contains two independent mol­ecules with slightly different conformations. In the crystal structure, weak inter­molecular C—H⋯O hydrogen bonds [C⋯O = 3.279 (15) and 3.407 (16) Å] link the mol­ecules into chains extended along the *c* axis.

## Related literature

For related crystal structures, see: Das *et al.* (1994 ▶); Huang *et al.* (2006 ▶). For general background, see: Bose *et al.* (2004 ▶).
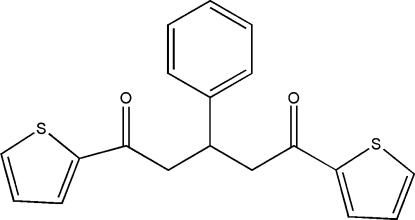


## Experimental

### 

#### Crystal data

C_19_H_16_O_2_S_2_
*M* *_r_* = 340.44
Orthorhombic, 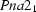

*a* = 27.912 (3) Å
*b* = 5.8607 (8) Å
*c* = 20.841 (2) Å
*V* = 3409.2 (7) Å^3^
*Z* = 8
Mo *K*α radiation
μ = 0.32 mm^−1^
*T* = 298 (2) K
0.26 × 0.17 × 0.09 mm

#### Data collection

Bruker SMART CCD area-detector diffractometer
Absorption correction: multi-scan (*SADABS*; Sheldrick, 1996 ▶) *T*_min_ = 0.922, *T*_max_ = 0.972
13190 measured reflections
5636 independent reflections
2006 reflections with *I* > 2σ(*I*)
*R*_int_ = 0.120

#### Refinement

*R*[*F* ^2^ > 2σ(*F* ^2^)] = 0.086
*wR*(*F* ^2^) = 0.192
*S* = 1.02
5636 reflections
415 parameters
1 restraint
H-atom parameters constrained
Δρ_max_ = 0.36 e Å^−3^
Δρ_min_ = −0.45 e Å^−3^
Absolute structure: Flack (1983 ▶), 2565 Friedel pairs
Flack parameter: 0.18 (15)

### 

Data collection: *SMART* (Siemens, 1996 ▶); cell refinement: *SAINT* (Siemens, 1996 ▶); data reduction: *SAINT*; program(s) used to solve structure: *SHELXS97* (Sheldrick, 2008 ▶); program(s) used to refine structure: *SHELXL97* (Sheldrick, 2008 ▶); molecular graphics: *SHELXTL* (Sheldrick, 2008 ▶); software used to prepare material for publication: *SHELXTL*.

## Supplementary Material

Crystal structure: contains datablocks I, global. DOI: 10.1107/S1600536808009884/cv2395sup1.cif

Structure factors: contains datablocks I. DOI: 10.1107/S1600536808009884/cv2395Isup2.hkl

Additional supplementary materials:  crystallographic information; 3D view; checkCIF report

## Figures and Tables

**Table 1:** Hydrogen-bond geometry (Å, °)

*D*—H⋯*A*	*D*—H	H⋯*A*	*D*⋯*A*	*D*—H⋯*A*
C21—H21⋯O4^i^	0.93	2.58	3.407 (16)	149
C1—H1⋯O2^ii^	0.93	2.54	3.279 (15)	137

Symmetry codes: (i) 
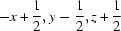
; (ii) 
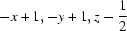
.

**Fig. 1.**
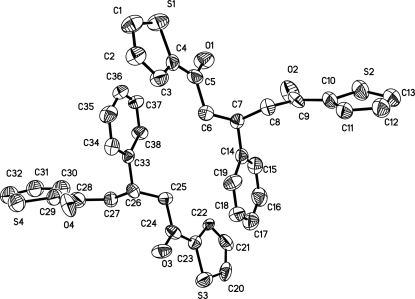
Drawing of the title compound with atomic numbering scheme and 30% probability displacement ellipsoids. Hydrogen atoms are omitted for clarity.

## supplementary crystallographic information

### Comment

In continuation of our ongoing program directed to the development of environmentally benign methods of chemical synthesis (Bose *et al.*, 2004), we have discovered a convenient one-step method for the preparation of 1,5-diketones starting from the fragrant aldehydes and fragrant ketones in the presence of NaOH under solvent-free conditions. Using this method, which can be considered as a general method for the synthesis of 1,5-diketones, we obtained the title compound, (I). We present here its crystal structure.

In (I) (Fig. 1), the asymmetric unit contains two independent molecules with slightly different conformations and normal bond lengths and angles comparable to those observed in 1,3,5-triphenyl-pentane-1,5-diketone (Das *et al.*, 1994) and 1,5-diphenyl-3-(2-pyridyl)pentane-1,5-dione (Huang *et al.*, 2006). The weak intermolecular C—H···O hydrogen bonds (Table 1) link the molecules into one-dimensional chains extending along the *c* axis.

### Experimental

2-Acetylthiophene (6.25 mmol) and freshly distilled benzaldehyde (3.125 mmol), NaOH (0.25 g, 6.25 mmol) were aggregated with glass paddle in an open flask. The resulting mixture was washed with water for several times for removing NaOH, and recrystalized from ethanol, and afforded the title compound as crystalline solid. Elemental analysis: calculated for C_19_H_16_O_2_S_2_: C 67.03, H 4.74%; Found: C 67.08, H 4.72%.

### Refinement

All H atoms were positioned geometrically and refined using a riding model with C-H = 0.93-0.98 Å and *U*_iso_(H) = *U*_eq_(C).

### Crystal data

**Table:**

C_19_H_16_O_2_S_2_	*D*_x_ = 1.327 Mg m^−^^3^
*M**_r_* = 340.44	Mo *K*α radiation λ = 0.71073 Å
Orthorhombic, *P**n**a*2_1_	Cell parameters from 1120 reflections
*a* = 27.912 (3) Å	θ = 2.9–25.0º
*b* = 5.8607 (8) Å	µ = 0.32 mm^−^^1^
*c* = 20.841 (2) Å	*T* = 298 (2) K
*V* = 3409.2 (7) Å^3^	Block, yellow
*Z* = 8	0.26 × 0.17 × 0.09 mm
*F*_000_ = 1424	

### Data collection

**Table:**

Bruker SMART CCD area-detector diffractometer	5636 independent reflections
Radiation source: fine-focus sealed tube	2006 reflections with *I* > 2σ(*I*)
Monochromator: graphite	*R*_int_ = 0.120
*T* = 298(2) K	θ_max_ = 25.0º
φ and ω scans	θ_min_ = 1.5º
Absorption correction: multi-scan(SADABS; Sheldrick, 1996)	*h* = −14→33
*T*_min_ = 0.922, *T*_max_ = 0.972	*k* = −6→6
13190 measured reflections	*l* = −24→20

### Refinement

**Table:**

Refinement on *F*^2^	Hydrogen site location: inferred from neighbouring sites
Least-squares matrix: full	H-atom parameters constrained
*R*[*F*^2^ > 2σ(*F*^2^)] = 0.086	*w* = 1/[σ^2^(*F*_o_^2^) + (0.0478*P*)^2^] where *P* = (*F*_o_^2^ + 2*F*_c_^2^)/3
*wR*(*F*^2^) = 0.192	(Δ/σ)_max_ = 0.001
*S* = 1.02	Δρ_max_ = 0.36 e Å^−^^3^
5636 reflections	Δρ_min_ = −0.45 e Å^−^^3^
415 parameters	Extinction correction: none
1 restraint	Absolute structure: Flack (1983), 2565 Friedel pairs
Primary atom site location: structure-invariant direct methods	Flack parameter: 0.18 (15)
Secondary atom site location: difference Fourier map	

### Special details

**Table:**

Geometry. All e.s.d.'s (except the e.s.d. in the dihedral angle between two l.s. planes) are estimated using the full covariance matrix. The cell e.s.d.'s are taken into account individually in the estimation of e.s.d.'s in distances, angles and torsion angles; correlations between e.s.d.'s in cell parameters are only used when they are defined by crystal symmetry. An approximate (isotropic) treatment of cell e.s.d.'s is used for estimating e.s.d.'s involving l.s. planes.
Refinement. Refinement of *F*^2^ against ALL reflections. The weighted *R*-factor *wR* and goodness of fit *S* are based on *F*^2^, conventional *R*-factors *R* are based on *F*, with *F* set to zero for negative *F*^2^. The threshold expression of *F*^2^ > σ(*F*^2^) is used only for calculating *R*-factors(gt) *etc*. and is not relevant to the choice of reflections for refinement. *R*-factors based on *F*^2^ are statistically about twice as large as those based on *F*, and *R*- factors based on ALL data will be even larger.

### Fractional atomic coordinates and isotropic or equivalent isotropic displacement parameters (Å^2^)

**Table:**

	*x*	*y*	*z*	*U*_iso_*/*U*_eq_	
O1	0.4895 (3)	0.4760 (15)	−0.0653 (3)	0.100 (3)	
O2	0.5431 (3)	0.5207 (16)	0.1105 (5)	0.117 (3)	
O3	0.2607 (3)	1.1103 (14)	0.1107 (3)	0.093 (2)	
O4	0.2020 (3)	0.8734 (18)	−0.0734 (4)	0.117 (3)	
S1	0.49589 (12)	0.5733 (5)	−0.20432 (17)	0.0981 (11)	
S2	0.62799 (13)	0.6688 (7)	0.18264 (19)	0.1168 (13)	
S3	0.25330 (11)	1.0432 (5)	0.24992 (17)	0.0937 (10)	
S4	0.11681 (15)	0.6173 (10)	−0.1171 (2)	0.1467 (19)	
C1	0.4854 (4)	0.772 (2)	−0.2610 (6)	0.091 (4)	
H1	0.4894	0.7482	−0.3048	0.109*	
C2	0.4704 (4)	0.969 (2)	−0.2348 (6)	0.081 (3)	
H2	0.4640	1.1001	−0.2585	0.098*	
C3	0.4655 (4)	0.957 (2)	−0.1689 (6)	0.078 (3)	
H3	0.4550	1.0777	−0.1436	0.094*	
C4	0.4779 (3)	0.747 (2)	−0.1452 (6)	0.064 (3)	
C5	0.4773 (4)	0.676 (2)	−0.0809 (6)	0.074 (3)	
C6	0.4575 (4)	0.8254 (19)	−0.0275 (5)	0.075 (3)	
H6A	0.4648	0.9830	−0.0378	0.089*	
H6B	0.4229	0.8103	−0.0276	0.089*	
C7	0.4748 (4)	0.7802 (17)	0.0391 (5)	0.058 (3)	
H7	0.4733	0.6147	0.0455	0.070*	
C8	0.5276 (3)	0.8490 (17)	0.0456 (5)	0.064 (3)	
H8A	0.5434	0.8245	0.0047	0.077*	
H8B	0.5293	1.0107	0.0552	0.077*	
C9	0.5549 (4)	0.718 (2)	0.0976 (6)	0.075 (4)	
C10	0.5955 (4)	0.820 (2)	0.1275 (5)	0.087 (4)	
C11	0.6126 (4)	1.045 (2)	0.1228 (6)	0.090 (4)	
H11	0.5995	1.1584	0.0970	0.108*	
C12	0.6526 (5)	1.072 (2)	0.1631 (7)	0.102 (4)	
H12	0.6699	1.2077	0.1654	0.123*	
C13	0.6638 (4)	0.890 (2)	0.1975 (6)	0.107 (4)	
H13	0.6890	0.8860	0.2268	0.128*	
C14	0.4456 (3)	0.8857 (19)	0.0911 (6)	0.061 (3)	
C15	0.4379 (4)	0.782 (2)	0.1486 (7)	0.078 (3)	
H15	0.4512	0.6389	0.1559	0.093*	
C16	0.4109 (4)	0.883 (3)	0.1972 (7)	0.095 (4)	
H16	0.4060	0.8063	0.2356	0.114*	
C17	0.3914 (5)	1.096 (3)	0.1884 (7)	0.100 (5)	
H17	0.3740	1.1666	0.2210	0.121*	
C18	0.3979 (4)	1.200 (2)	0.1324 (8)	0.088 (4)	
H18	0.3832	1.3399	0.1258	0.105*	
C19	0.4253 (4)	1.1110 (19)	0.0834 (6)	0.083 (3)	
H19	0.4307	1.1945	0.0462	0.100*	
C20	0.2648 (4)	0.857 (3)	0.3085 (5)	0.099 (4)	
H20	0.2570	0.8801	0.3514	0.119*	
C21	0.2870 (4)	0.667 (2)	0.2862 (7)	0.094 (4)	
H21	0.2972	0.5507	0.3135	0.113*	
C22	0.2936 (3)	0.6553 (17)	0.2214 (4)	0.049 (3)	
H22	0.3067	0.5334	0.1988	0.058*	
C23	0.2767 (3)	0.8648 (17)	0.1944 (5)	0.059 (3)	
C24	0.2767 (3)	0.925 (2)	0.1271 (6)	0.067 (3)	
C25	0.2983 (4)	0.7695 (18)	0.0785 (5)	0.069 (3)	
H25A	0.3320	0.8066	0.0746	0.083*	
H25B	0.2961	0.6145	0.0946	0.083*	
C26	0.2767 (3)	0.7758 (17)	0.0135 (5)	0.060 (3)	
H26	0.2717	0.9366	0.0024	0.072*	
C27	0.2264 (4)	0.6603 (19)	0.0151 (5)	0.072 (3)	
H27A	0.2310	0.4973	0.0203	0.087*	
H27B	0.2095	0.7148	0.0527	0.087*	
C28	0.1956 (5)	0.698 (3)	−0.0415 (7)	0.090 (4)	
C29	0.1564 (5)	0.554 (4)	−0.0595 (8)	0.125 (6)	
C30	0.1481 (6)	0.341 (4)	−0.0319 (8)	0.141 (7)	
H30	0.1666	0.2724	0.0000	0.169*	
C31	0.1071 (6)	0.247 (3)	−0.0608 (8)	0.141 (7)	
H31	0.0953	0.1058	−0.0480	0.169*	
C32	0.0852 (6)	0.368 (3)	−0.1074 (9)	0.144 (7)	
H32	0.0581	0.3241	−0.1302	0.173*	
C33	0.3080 (3)	0.6706 (19)	−0.0394 (5)	0.054 (3)	
C34	0.3164 (4)	0.7746 (18)	−0.0964 (6)	0.066 (3)	
H34	0.3017	0.9139	−0.1048	0.079*	
C35	0.3456 (5)	0.683 (3)	−0.1420 (6)	0.086 (4)	
H35	0.3525	0.7628	−0.1793	0.103*	
C36	0.3646 (4)	0.470 (3)	−0.1320 (7)	0.098 (4)	
H36	0.3828	0.4010	−0.1638	0.117*	
C37	0.3570 (4)	0.364 (2)	−0.0771 (7)	0.088 (4)	
H37	0.3719	0.2247	−0.0703	0.106*	
C38	0.3279 (4)	0.4499 (18)	−0.0293 (5)	0.071 (3)	
H38	0.3216	0.3671	0.0078	0.086*	

### Atomic displacement parameters (Å^2^)

**Table:**

	*U*^11^	*U*^22^	*U*^33^	*U*^12^	*U*^13^	*U*^23^
O1	0.139 (7)	0.062 (6)	0.100 (6)	0.030 (5)	−0.003 (5)	−0.002 (5)
O2	0.103 (7)	0.085 (7)	0.163 (8)	−0.004 (5)	−0.025 (6)	0.055 (6)
O3	0.127 (7)	0.055 (6)	0.096 (6)	−0.017 (5)	−0.016 (5)	−0.007 (5)
O4	0.072 (6)	0.154 (10)	0.125 (8)	0.003 (6)	0.026 (5)	0.043 (7)
S1	0.127 (3)	0.068 (2)	0.100 (2)	0.006 (2)	0.014 (2)	−0.017 (2)
S2	0.098 (3)	0.120 (3)	0.133 (3)	0.028 (2)	−0.036 (2)	−0.004 (3)
S3	0.102 (2)	0.085 (2)	0.094 (2)	0.001 (2)	−0.003 (2)	−0.020 (2)
S4	0.077 (3)	0.214 (5)	0.149 (4)	−0.018 (3)	0.024 (3)	−0.068 (4)
C1	0.115 (10)	0.074 (10)	0.083 (10)	−0.017 (8)	0.020 (8)	−0.015 (8)
C2	0.108 (9)	0.056 (9)	0.080 (10)	0.004 (7)	0.004 (8)	0.009 (7)
C3	0.091 (9)	0.071 (10)	0.072 (9)	0.007 (7)	0.003 (7)	−0.005 (8)
C4	0.046 (6)	0.090 (11)	0.056 (8)	−0.007 (6)	−0.005 (6)	−0.001 (7)
C5	0.063 (8)	0.059 (8)	0.100 (10)	0.015 (6)	−0.009 (7)	−0.021 (8)
C6	0.074 (7)	0.073 (8)	0.076 (9)	0.013 (6)	−0.008 (7)	−0.011 (7)
C7	0.068 (8)	0.047 (7)	0.060 (7)	−0.002 (5)	−0.008 (7)	0.000 (5)
C8	0.062 (8)	0.065 (8)	0.066 (7)	−0.005 (6)	−0.004 (6)	0.011 (6)
C9	0.042 (7)	0.089 (10)	0.094 (10)	0.010 (7)	0.009 (7)	0.017 (8)
C10	0.067 (9)	0.085 (10)	0.109 (10)	0.023 (8)	−0.022 (7)	−0.013 (8)
C11	0.077 (9)	0.083 (10)	0.110 (10)	0.016 (8)	−0.016 (8)	−0.022 (8)
C12	0.091 (10)	0.088 (11)	0.128 (12)	0.010 (8)	−0.020 (9)	−0.017 (9)
C13	0.087 (10)	0.107 (12)	0.125 (11)	0.026 (9)	−0.024 (8)	−0.023 (10)
C14	0.056 (7)	0.054 (8)	0.072 (9)	−0.004 (6)	−0.015 (6)	−0.004 (7)
C15	0.083 (9)	0.065 (8)	0.085 (10)	−0.002 (7)	−0.014 (8)	0.005 (8)
C16	0.068 (9)	0.124 (14)	0.094 (11)	−0.019 (9)	0.000 (8)	0.003 (10)
C17	0.076 (9)	0.149 (16)	0.077 (11)	−0.008 (10)	0.008 (8)	−0.025 (11)
C18	0.076 (9)	0.088 (10)	0.099 (11)	0.009 (7)	−0.006 (8)	−0.032 (10)
C19	0.105 (10)	0.051 (9)	0.093 (9)	−0.005 (7)	0.002 (8)	−0.018 (7)
C20	0.076 (8)	0.176 (15)	0.046 (8)	0.022 (9)	0.006 (6)	−0.008 (9)
C21	0.073 (8)	0.087 (10)	0.123 (13)	−0.017 (7)	0.022 (8)	0.012 (10)
C22	0.067 (7)	0.053 (7)	0.026 (6)	−0.008 (5)	0.001 (5)	0.002 (5)
C23	0.052 (6)	0.074 (8)	0.050 (7)	0.013 (5)	−0.011 (6)	−0.003 (6)
C24	0.054 (7)	0.048 (8)	0.098 (10)	−0.002 (6)	−0.006 (6)	0.010 (8)
C25	0.074 (8)	0.069 (8)	0.064 (8)	−0.016 (6)	−0.002 (7)	−0.011 (6)
C26	0.052 (7)	0.057 (7)	0.069 (8)	−0.001 (5)	0.004 (6)	0.013 (6)
C27	0.066 (8)	0.084 (9)	0.067 (8)	−0.001 (6)	0.004 (6)	0.002 (6)
C28	0.091 (10)	0.103 (11)	0.076 (10)	−0.019 (9)	−0.007 (8)	0.008 (8)
C29	0.057 (10)	0.182 (19)	0.134 (15)	0.002 (12)	0.018 (10)	−0.077 (14)
C30	0.078 (12)	0.18 (2)	0.163 (17)	0.000 (13)	0.018 (11)	−0.081 (15)
C31	0.079 (13)	0.19 (2)	0.159 (17)	−0.002 (12)	0.017 (11)	−0.078 (15)
C32	0.080 (12)	0.19 (2)	0.161 (17)	0.004 (12)	0.023 (11)	−0.088 (14)
C33	0.046 (6)	0.064 (8)	0.052 (7)	0.011 (5)	0.002 (5)	−0.006 (6)
C34	0.075 (8)	0.059 (8)	0.063 (9)	0.015 (6)	0.004 (7)	0.015 (6)
C35	0.091 (10)	0.109 (13)	0.058 (9)	0.012 (8)	−0.020 (8)	0.005 (8)
C36	0.085 (9)	0.134 (16)	0.074 (10)	−0.019 (10)	0.004 (8)	−0.043 (10)
C37	0.092 (10)	0.084 (10)	0.088 (10)	−0.024 (8)	0.012 (9)	−0.039 (10)
C38	0.093 (8)	0.039 (7)	0.083 (9)	−0.013 (6)	0.001 (7)	−0.002 (6)

### Geometric parameters (Å, °)

**Table:**

O1—C5	1.263 (12)	C16—H16	0.9300
O2—C9	1.232 (12)	C17—C18	1.328 (16)
O3—C24	1.225 (11)	C17—H17	0.9300
O4—C28	1.239 (13)	C18—C19	1.376 (14)
S1—C4	1.674 (11)	C18—H18	0.9300
S1—C1	1.683 (13)	C19—H19	0.9300
S2—C13	1.667 (14)	C20—C21	1.356 (15)
S2—C10	1.712 (11)	C20—H20	0.9300
S3—C20	1.671 (13)	C21—C22	1.364 (13)
S3—C23	1.692 (10)	C21—H21	0.9300
S4—C29	1.672 (17)	C22—C23	1.430 (12)
S4—C32	1.72 (2)	C22—H22	0.9300
C1—C2	1.346 (14)	C23—C24	1.445 (14)
C1—H1	0.9300	C24—C25	1.488 (14)
C2—C3	1.380 (12)	C25—C26	1.484 (12)
C2—H2	0.9300	C25—H25A	0.9700
C3—C4	1.373 (13)	C25—H25B	0.9700
C3—H3	0.9300	C26—C33	1.536 (13)
C4—C5	1.404 (15)	C26—C27	1.557 (12)
C5—C6	1.520 (14)	C26—H26	0.9800
C6—C7	1.493 (13)	C27—C28	1.477 (16)
C6—H6A	0.9700	C27—H27A	0.9700
C6—H6B	0.9700	C27—H27B	0.9700
C7—C14	1.489 (13)	C28—C29	1.433 (19)
C7—C8	1.534 (12)	C29—C30	1.39 (2)
C7—H7	0.9800	C30—C31	1.41 (2)
C8—C9	1.530 (14)	C30—H30	0.9300
C8—H8A	0.9700	C31—C32	1.35 (2)
C8—H8B	0.9700	C31—H31	0.9300
C9—C10	1.425 (15)	C32—H32	0.9300
C10—C11	1.405 (15)	C33—C34	1.357 (13)
C11—C12	1.408 (15)	C33—C38	1.423 (13)
C11—H11	0.9300	C34—C35	1.362 (14)
C12—C13	1.323 (15)	C34—H34	0.9300
C12—H12	0.9300	C35—C36	1.368 (16)
C13—H13	0.9300	C35—H35	0.9300
C14—C15	1.361 (14)	C36—C37	1.318 (15)
C14—C19	1.446 (14)	C36—H36	0.9300
C15—C16	1.394 (15)	C37—C38	1.379 (14)
C15—H15	0.9300	C37—H37	0.9300
C16—C17	1.374 (17)	C38—H38	0.9300
			
C4—S1—C1	92.5 (6)	C18—C19—H19	120.6
C13—S2—C10	92.3 (7)	C14—C19—H19	120.6
C20—S3—C23	91.2 (6)	C21—C20—S3	112.0 (10)
C29—S4—C32	93.7 (10)	C21—C20—H20	124.0
C2—C1—S1	111.3 (10)	S3—C20—H20	124.0
C2—C1—H1	124.3	C20—C21—C22	116.1 (13)
S1—C1—H1	124.3	C20—C21—H21	121.9
C1—C2—C3	113.0 (12)	C22—C21—H21	121.9
C1—C2—H2	123.5	C21—C22—C23	107.7 (10)
C3—C2—H2	123.5	C21—C22—H22	126.2
C4—C3—C2	112.2 (11)	C23—C22—H22	126.2
C4—C3—H3	123.9	C22—C23—C24	126.2 (9)
C2—C3—H3	123.9	C22—C23—S3	112.8 (8)
C3—C4—C5	127.3 (11)	C24—C23—S3	120.9 (8)
C3—C4—S1	110.9 (9)	O3—C24—C23	119.1 (11)
C5—C4—S1	121.8 (10)	O3—C24—C25	120.0 (11)
O1—C5—C4	121.0 (11)	C23—C24—C25	120.7 (10)
O1—C5—C6	116.4 (12)	C26—C25—C24	116.2 (10)
C4—C5—C6	122.3 (11)	C26—C25—H25A	108.2
C7—C6—C5	117.4 (10)	C24—C25—H25A	108.2
C7—C6—H6A	107.9	C26—C25—H25B	108.2
C5—C6—H6A	107.9	C24—C25—H25B	108.2
C7—C6—H6B	107.9	H25A—C25—H25B	107.4
C5—C6—H6B	107.9	C25—C26—C33	114.4 (9)
H6A—C6—H6B	107.2	C25—C26—C27	109.6 (8)
C14—C7—C6	115.2 (9)	C33—C26—C27	110.7 (8)
C14—C7—C8	110.6 (9)	C25—C26—H26	107.3
C6—C7—C8	110.2 (9)	C33—C26—H26	107.3
C14—C7—H7	106.8	C27—C26—H26	107.3
C6—C7—H7	106.8	C28—C27—C26	116.3 (10)
C8—C7—H7	106.8	C28—C27—H27A	108.2
C9—C8—C7	114.1 (9)	C26—C27—H27A	108.2
C9—C8—H8A	108.7	C28—C27—H27B	108.2
C7—C8—H8A	108.7	C26—C27—H27B	108.2
C9—C8—H8B	108.7	H27A—C27—H27B	107.4
C7—C8—H8B	108.7	O4—C28—C29	117.4 (15)
H8A—C8—H8B	107.6	O4—C28—C27	117.9 (13)
O2—C9—C10	120.7 (11)	C29—C28—C27	124.6 (15)
O2—C9—C8	119.5 (11)	C30—C29—C28	123.0 (19)
C10—C9—C8	119.6 (11)	C30—C29—S4	112.8 (14)
C11—C10—C9	129.3 (11)	C28—C29—S4	124.1 (17)
C11—C10—S2	110.6 (10)	C29—C30—C31	108 (2)
C9—C10—S2	119.9 (10)	C29—C30—H30	126.1
C10—C11—C12	109.5 (12)	C31—C30—H30	126.1
C10—C11—H11	125.2	C32—C31—C30	118 (2)
C12—C11—H11	125.2	C32—C31—H31	120.9
C13—C12—C11	114.8 (14)	C30—C31—H31	120.9
C13—C12—H12	122.6	C31—C32—S4	107.4 (15)
C11—C12—H12	122.6	C31—C32—H32	126.3
C12—C13—S2	112.7 (11)	S4—C32—H32	126.3
C12—C13—H13	123.7	C34—C33—C38	118.0 (10)
S2—C13—H13	123.7	C34—C33—C26	123.2 (10)
C15—C14—C19	116.2 (11)	C38—C33—C26	118.8 (10)
C15—C14—C7	122.8 (11)	C33—C34—C35	122.4 (11)
C19—C14—C7	120.9 (11)	C33—C34—H34	118.8
C14—C15—C16	122.4 (12)	C35—C34—H34	118.8
C14—C15—H15	118.8	C34—C35—C36	119.1 (13)
C16—C15—H15	118.8	C34—C35—H35	120.5
C17—C16—C15	120.3 (14)	C36—C35—H35	120.5
C17—C16—H16	119.9	C37—C36—C35	119.9 (14)
C15—C16—H16	119.9	C37—C36—H36	120.1
C18—C17—C16	118.4 (14)	C35—C36—H36	120.1
C18—C17—H17	120.8	C36—C37—C38	123.3 (14)
C16—C17—H17	120.8	C36—C37—H37	118.3
C17—C18—C19	123.8 (13)	C38—C37—H37	118.3
C17—C18—H18	118.1	C37—C38—C33	117.0 (11)
C19—C18—H18	118.1	C37—C38—H38	121.5
C18—C19—C14	118.7 (12)	C33—C38—H38	121.5

### Hydrogen-bond geometry (Å, °)

**Table:**

*D*—H···*A*	*D*—H	H···*A*	*D*···*A*	*D*—H···*A*
C21—H21···O4^i^	0.93	2.58	3.407 (16)	149
C1—H1···O2^ii^	0.93	2.54	3.279 (15)	137

Symmetry codes: (i) −*x*+1/2, *y*−1/2, *z*+1/2; (ii) −*x*+1, −*y*+1, *z*−1/2.

## References

[bb1] Bose, A. K., Pednekar, S., Ganguly, S. N., Chakraborty, G. & Manhas, M. S. (2004). *Tetrahedron Lett.* **45**, 8351–8353.

[bb2] Das, G. C., Hursthouse, M. B., Malik, K. M. A., Rahman, M. M., Rahman, M. T. & Olsson, T. (1994). *J. Chem. Crystallogr.* **24**, 511–515.

[bb3] Flack, H. D. (1983). *Acta Cryst.* A**39**, 876–881.

[bb4] Huang, X.-Q., Wang, D.-Q., Dou, J.-M. & Wang, J.-X. (2006). *Acta Cryst.* E**62**, o60–o61.

[bb5] Sheldrick, G. M. (1996). *SADABS* University of Göttingen, Germany.

[bb6] Sheldrick, G. M. (2008). *Acta Cryst.* A**64**, 112–122. 10.1107/S0108767307043930 18156677

[bb7] Siemens (1996). *SMART* and *SAINT* Siemens Analytical X-ray Instruments Inc., Madison, Wisconsin, USA.

